# Stable fixation of an osseointegated implant system for above-the-knee amputees: Titel RSA and radiographic evaluation of migration and bone remodeling in 55 cases

**DOI:** 10.3109/17453674.2012.678799

**Published:** 2012-04-24

**Authors:** Audrey Nebergall, Charles Bragdon, Anne Antonellis, Johan Kärrholm, Rickard Brånemark, Henrik Malchau

**Affiliations:** ^1^Harris Orthopaedic Laboratory, Massachusetts General Hospital, Boston, MA, USA; ^2^Sahlgrenska University Hospital, Göteborg, Sweden.

## Abstract

**Background and purpose:**

Rehabilitation of patients with transfemoral amputations is particularly difficult due to problems in using standard socket prostheses. We wanted to assess long-term fixation of the osseointegrated implant system (OPRA) using radiostereometric analysis (RSA) and periprosthetic bone remodeling.

**Methods:**

51 patients with transfemoral amputations (55 implants) were enrolled in an RSA study. RSA and plain radiographs were scheduled at 6 months and at 1, 2, 5, 7, and 10 years after surgery. RSA films were analyzed using UmRSA software. Plain radiographs were graded for bone resorption, cancellization, cortical thinning, and trabecular streaming or buttressing in specifically defined zones around the implant.

**Results:**

At 5 years, the median (SE) migration of the implant was –0.02 (0.06) mm distally. The rotational movement was 0.42 (0.32) degrees around the longitudinal axis. There was no statistically significant difference in median rotation or migration at any follow-up time. Cancellization of the cortex (plain radiographic grading) appeared in at least 1 zone in over half of the patients at 2 years. However, the prevalence of cancellization had decreased by the 5-year follow-up.

**Interpretation:**

The RSA analysis for the OPRA system indicated stable fixation of the implant. The periprosthetic bone remodeling showed similarities with changes seen around uncemented hip stems. The OPRA system is a new and promising approach for addressing the challenges faced by patients with transfemoral amputations.

The traditional method of attaching prostheses for patients who have undergone a transfemoral amputation is by means of socket prostheses. Numerous studies have documented the shortcomings of this approach (Hoaglund et al. 1983, Hagberg and Branemark 2001, Hoffman et al. 2002). Skin conditions and volume changes of the stump increase the difficulty in properly attaching and using the prosthesis (Sherman 1999, Collins et al. 2006). In addition, patients experience changes in gait, which reduces hip flexion and extension and increases pelvic tilt (Hagberg et al. 2005, Rabuffetti et al. 2005). There is also a lack of stabilization between the prosthesis and the residual limb.

Brånemark introduced the concept of osseointegration in the 1950s (Sullivan 2001). Shortly afterwards, it was applied to human dental implants. Osseointegration was originally defined as “a direct structural and functional connection between ordered living bone and the surface of a load-carrying implant” (Branemark et al. 1977, 2001). This definition has since been modified over the years as follows: “...when there is no progressive relative movement between the implant and the bone with which it has direct contact” (Branemark et al. 2001). Adell et al. (1981) maintained that the fundamental crux and success of osseointegration was threefold: a delicate surgical technique, an adequate recovery period to allow for optimal bony ingrowth, and controlled loading when use of the implant begins. The bone remodeling that occurs around an osseointegrated implant during the carefully controlled rehabilitation permits further integration of the implant into the bone and gives enhanced long-term clinical outcome. The importance of osseointegration for the long-term stability of orthopedic implants has been successfully used in total joint arthroplasty (Engh et al. 2003, Glassman et al. 2006). This approach may provide a stable and functional prosthesis for patients who cannot use a conventional socket prosthesis (Hagberg and Branemark 2009).

A number of studies have examined the positive effect of osseointegrated prostheses on the skin around the site and also on joint movement relative to the effect of standard socket prostheses (Hagberg et al. 2005, Lee et al. 2007, Hagberg and Branemark 2009). Increased quality of life, overall well-being, and improved prosthetic usage have been reported (Hagberg et al. 2008).

We assessed long-term fixation and stability of the osseointegrated implant using radiostereometric analysis (RSA) and periprosthetic bone remodeling on plain radiographs. Our hypothesis was that there is not substantial micromotion of the implant and that periprosthetic bone remodeling does not have a negative effect on implant stability or performance.

## Patients and methods

51 patients with transfemoral amputations (mean age at implant surgery: 45 (21–65) years; 28 males) were enrolled in the prospective OPRA (Osseointegrated Prostheses for the Rehabilitation of Amputees) study. There were 45 unilateral patients and 6 bilateral patients. 2 bilateral patients were only treated on one side in this study; thus, 55 prosthetic systems were enrolled and followed in this study. Most patients had had a traumatic injury or a tumor.

**Figure 1.**
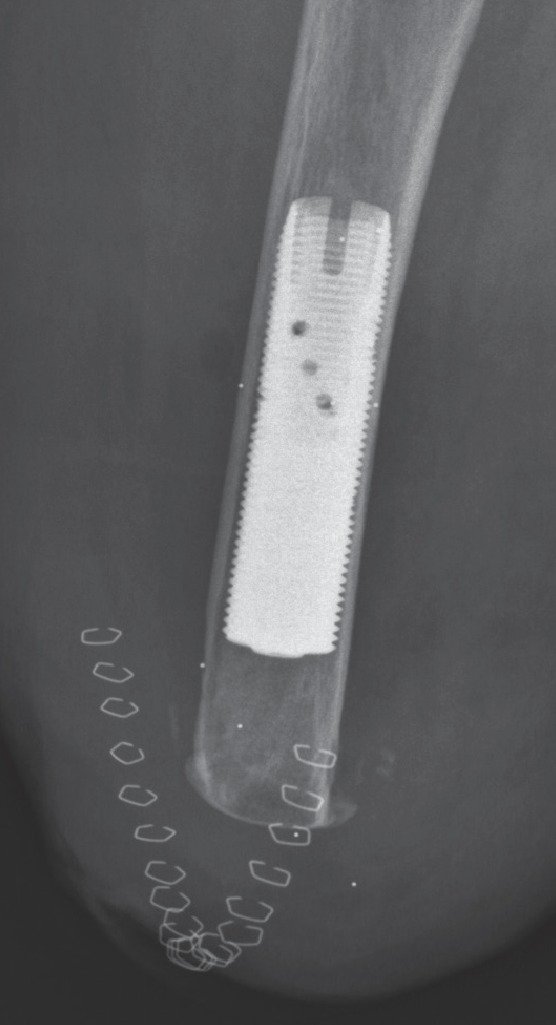
Implant after stage-2 (S1) surgery with tantalum RSA beads.

**Figure 2.**
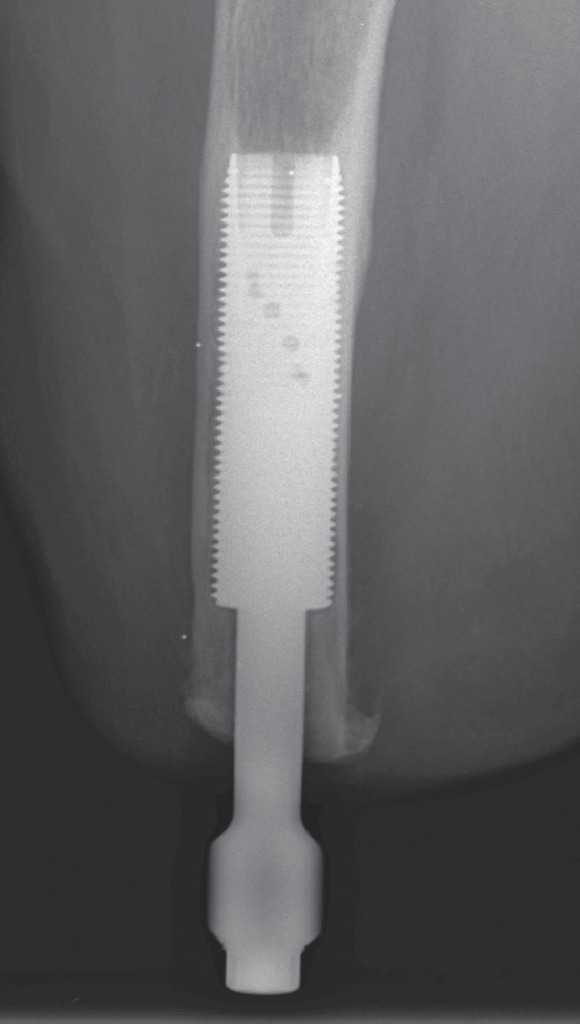
Implant with the addition of the transcutaneous abutment after stage-2 (S2) surgery.

The surgeries were performed at Sahlgrenska University Hospital in Sweden. The OPRA Implant System was implanted in a 2-stage surgical procedure. The first-stage surgeries (S1) were performed between May 1999 and December 2007. At the first stage, a threaded titanium implant (fixture) was inserted into the remaining distal femur. Specific instruments were used that permit precise sizing and optimal positioning of the implant into the distal femoral canal. The fixture was pre-marked with 6 tantalum RSA beads, and 6–8 tantalum beads were placed in the femoral cortical bone surrounding the implant (Figure 1). After 6 months of unloading, or loading with a conventional socket prosthesis, during which the implant becomes osseointegrated into the bone, a second surgery (S2) was done to attach the transcutaneous abutment to the femoral fixture (Figure 2). This abutment provided attachment for an external, removable prosthesis after a rehabilitation program had been initiated. 

The OPRA study protocol has several efficacy parameters and also reporting of adverse events. This paper concerns the RSA and plain radiographic part of the study. Plain and RSA radiographic follow-up was planned for 6 months and for 1, 2, 5, 7, and 10 years after the second-stage surgery. All films taken prior to 2005 were analog. However, all analog films were scanned and analyzed as digital images in UmRSA.

### Radiostereometric analysis

**Figure 3.**
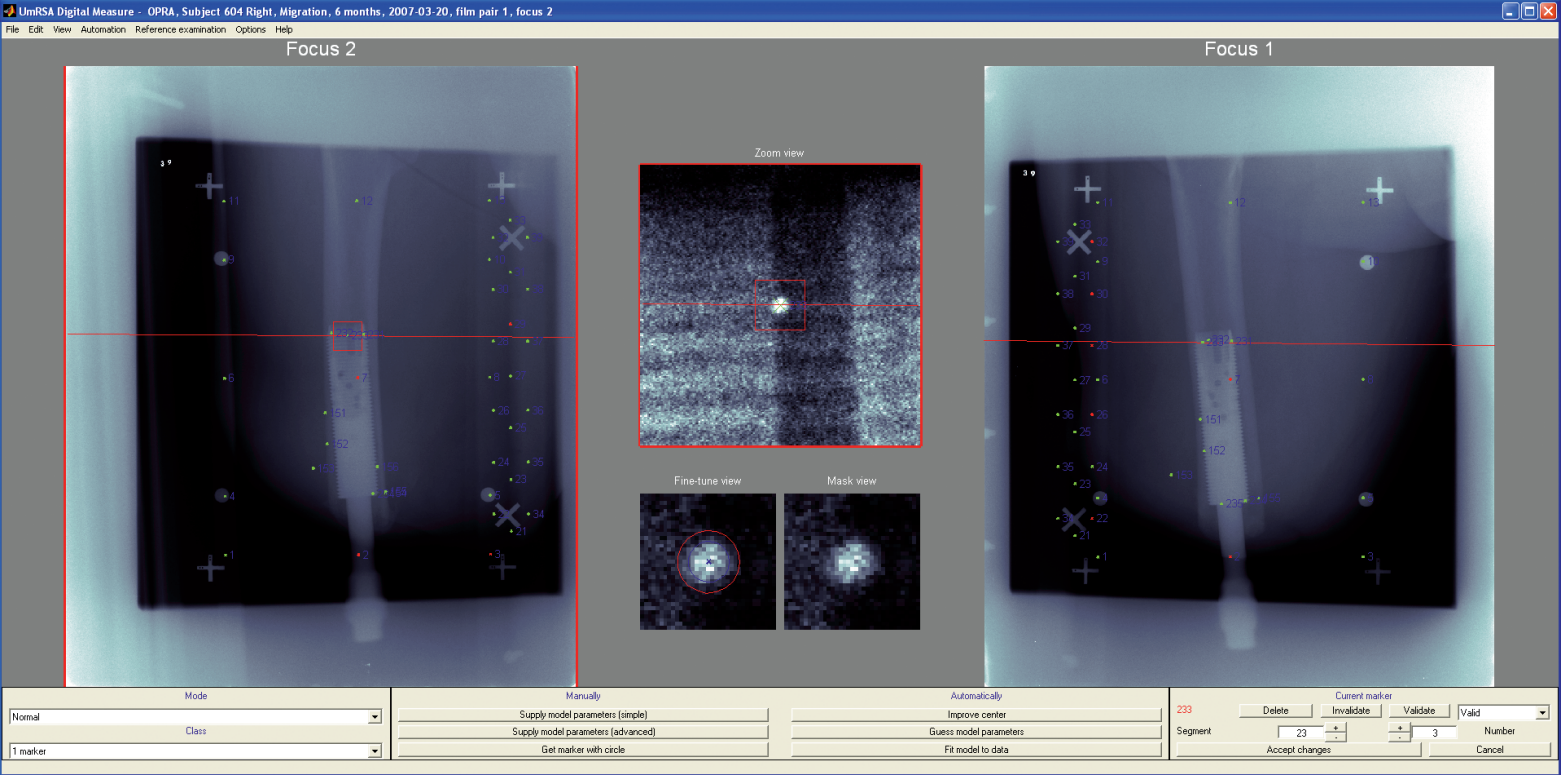
The UmRSA 6.0 software interface with cage beads, implant beads, and femur beads marked in green and red. An implant bead is selected and zoomed in on in the middle of the screen.

**Figure 4.**
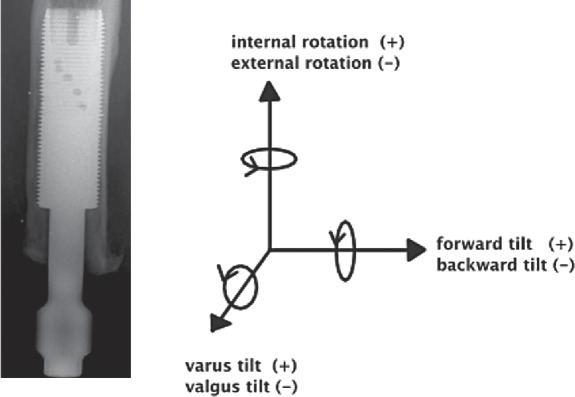
The defined directions of rotation of the implant.

The accuracy of the RSA system for evaluating implant micromotion early in follow-up is approximately 50 μm (Bragdon et al. 2004). Duplicate examinations would be necessary to determine the accuracy of the set-up in the current study, but these were not performed. RSA examinations of femoral stems were done during the same time period, at the same RSA laboratory, and are referred to as the “double examinations” of our study. Thien et al. (2010) estimated the 99% confidence limits for the error concerning proximal/distal translation and rotations around the transverse, longitudinal, and sagittal axes to be 0.22 mm and 0.47, 0.96, and 0.26 degrees. The patients' films were analyzed using UmRSA software version 6.0 (RSA Biomedical, Umeå, Sweden) (Figure 3). A uniplanar calibration cage was positioned under the table and the patient was maneuvered so that the femur was centered within the reference points on the cage in both foci. The patient was oriented such that the femur and implant were parallel to the y-axis. This calibration cage served as a reference for the subsequent image comparisons; 2 stereoradiographs were taken simultaneously, and the 3D coordinates of the tantalum beads were determined. The femur and implant segments were defined by at least 3 beads in either region. Relative motion between the implant and bone segments was then calculated between serial radiographs. Using the 6-month film (after S2) as a baseline, the movement of the fixture relative to the femur was compared in all the follow-up films to determine whether there was any rotation or migration of the implant within the femur over time. Implant migration in the cranial direction was defined as positive motion—and conversely, migration in the caudal direction was defined as negative motion. The mean error (ME) limit was set at 0.25. Translations (migration) were measured at the gravitational center of the markers inserted into the fixture. We measured rotations as forward(+)/backward(–) tilting, internal(+)/external(–) rotation, and varus (+)/valgus(–) angulation corresponding to rotations around the transverse (x-), the longitudinal (y-) and sagittal (z-) axes (Figure 4).

### Plain radiographs (Figure 6)

**Figure 5.**
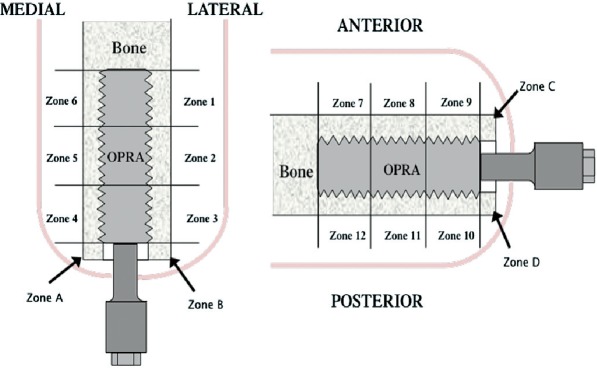
The bone surrounding the implant is divided into zones 1–6, A, and B for medial and lateral assessment of bone remodeling and zones 7–12, C, and D for anterior and posterior assessment of bone remodeling.

The bone surrounding the fixture was divided into 16 zones: A–D and 1–12 (Figure 5). Each follow-up plain radiograph was graded in 5 categories in the appropriate zones: distal bone resorption, endosteal bone resorption, cortical thinning, cancellization, and trabecular streaming or buttressing. Distal bone resorption is resorption of the distal bone, causing exposure of the femoral fixture. Endosteal resorption is resorption of the bone around the fixture with a radiolucent zone that is wider than the fixture thread depth. Cortical thinning is a decrease in the width of the cortex along the area of the bone where the fixture is implanted. Cancellization is an increase in the porosity of the cortex surrounding the fixture. Trabecular streaming or buttressing is defined as an increase in trabecular density at the proximal end of the implant, forming an angle between the inner cortex and the implant. If the zone had any evidence of these 5 types of remodeling compared to the S1 postoperative film, that zone was graded with a 1 in the appropriate category. If the zone had no evidence of any type of remodeling, that zone was graded as 0.

**Figure 6.**
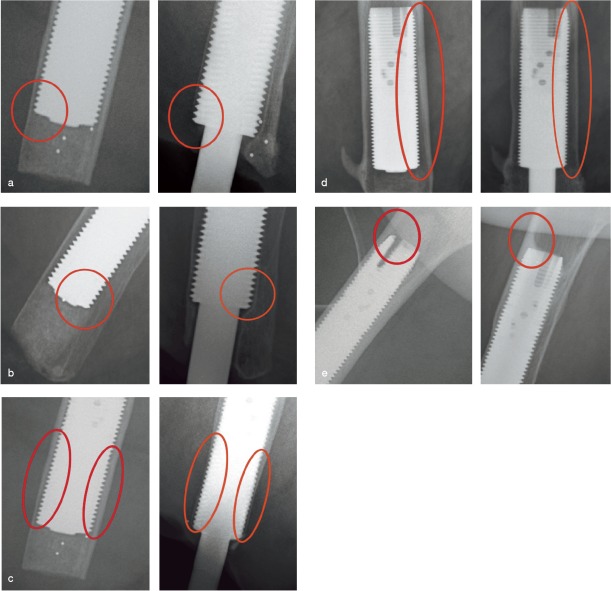
Distal bone resorption: zones A–D. Endosteal resorption: zones 1–12. Cortical thinning: zones 1–12. Cancellization: zones A–D and 1–12. Trabecular streaming or buttressing: zones 1, 6, 7, and 12. The stage-1 postoperative film shows the bone immediately following implantation of the device. Each 2-year film shows an example of distal bone resorption (panel a), endosteal resorption (b), cortical thinning (c), cancellization (d), and trabecular streaming or buttressing (e).

## Results

All patients in the OPRA study passed the 2-year follow-up after S2 and clinical details will be presented in a separate paper. Of the 55 implants in the study, the following numbers of implants were analyzed with RSA at each follow-up interval: 47 implants at 6 months, 42 implants at 1 year, 40 implants at 2 years, 15 implants at 5 years, 12 implants at 7 years, and 3 implants at 10 years. Due to the low number of implants with a 10-year follow-up, the 10-year data are not reported at this time. 8 patients were excluded from the study and 4 had their implants removed; 3 implants were removed due to loosening and 1 was removed due to infection. 1 patient did not have his implant removed but was excluded due to complications that were not from surgery, 1 patient was lost to follow-up, and 2 patients died. Only 2 of the 4 failed implants had RSA films analyzed at 1 and 2 years of follow-up. 1 patient did not receive any tantalum beads and did not therefore participate in the RSA part of the study (Table 1).

**Table 1.** Reason for exclusion from RSA analysis and patient count at each interval

Reason for exclusion from RSA analysis	Patient count at each interval			
	1 year	2 years	5 years	7 years
Patients eligible for follow-up	54	48	25	16
RSA follow-up complications **^a^**	10	6	10	4
Implant removed	2	2	–	–
Patients analyzed	42	40	15	12

**^a^** Complications included: misplaced analog films, lost to follow-up/missed follow-up, and analysis failures in UmRSA.

**Figure 7.**
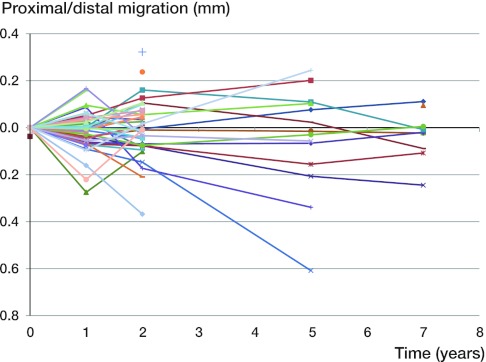
Individual proximal/distal migration (in mm) of the OPRA implants over time.

**Figure 8.**
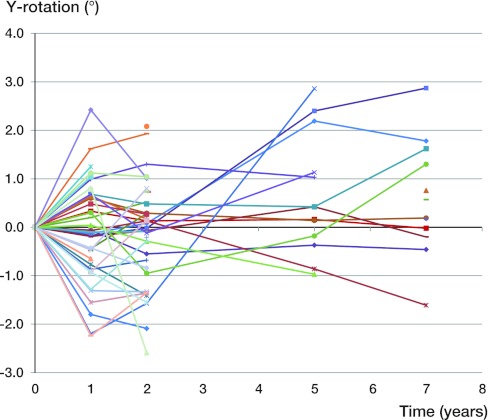
Individual rotation (in degrees) about the y-axis—the long axis of the femur—of the OPRA implants over time.

The median (SE) of the proximal/distal migration of the implant was –0.01 (0.01) mm at 1 year, –0.01 (0.02) mm at 2 years, –0.02 (0.06) at 5 years, and –0.02 (0.03) mm distally at 7 years (Table 2). The median reference segment mean error (ME) for all the films over time was 0.17 (0.04–0.25) and the median current segment ME was 0.15 (0.02–0.25). The median reference segment condition number (CN) was 53 (25–395); 4 films had a condition number above 200 and in all cases only 3 or 4 beads were visible. None of these 4 films showed a high ME (0.069–0.213). The median current segment condition number was 59 (51–120). The median (SE) of the rotational movement around the longitudinal axis was –0.10 (0.16) degrees at 1 year, –0.08 (0.17) degrees at 2 years, 0.42 (0.32) degrees at 5 years, and 0.38 (0.34) degrees at 7 years. The greatest median rotational movement occurred along the longitudinal axis at all follow-up time points (Table 3). The individual RSA analyses suggested that there was no pattern of increased proximal/distal migration over time. The confidence intervals show that there was no certain migration of the entire cohort of observations in either the proximal direction or the distal direction at a minimum of 5 years of follow-up (Table 2 and Figure 7). A similar trend was seen with the rotations, as there was no significant rotation in the entire cohort (Table 3 and Figure 8). A Mann-Whitney non-parametric test and a Wilcoxon signed-rank test showed that there was no statistically significant difference in the median rotations or proximal/distal migrations at any follow-up interval (p > 0.05).

**Table 2.** Proximal/distal migration (in mm) of the implant at 1, 2, 5, and 7 years of follow-up

	Proximal/distal (y-axis) migration (mm)			
	1 year	2 years	5 years	7 years
Mean	–0.01	0.00	–0.05	–0.03
Median	–0.01	–0.01	–0.02	–0.02
Standard error	0.01	0.02	0.06	0.03
Minimum	–0.28	–0.37	–0.61	–0.25
Maximum	0.17	0.32	0.24	0.11
Count	42	40	15	12
Lower 95% CI	–0.37	–0.53	0.09	–0.09
Upper 95% CI	0.25	0.13	1.33	1.25

**Table 3.** Rotation about the x–, y–, and z–axes (in degrees) of the implant at 1, 2, 5, and 7 years of follow–up

	Rotation (degrees)											
	1 year	1 year	1 year	2 years	2 years	2 years	5 years	5 years	5 years	7 years	7 years	7 years
	x-axis	y-axis	z-axis	x-axis	y-axis	z-axis	x-axis	y-axis	z-axis	x-axis	y-axis	z-axis
Mean	–0.07	–0.06	–0.03	–0.01	–0.20	0.04	–0.32	0.71	–0.04	0.08	0.58	–0.02
Median	–0.09	–0.10	–0.04	0.02	–0.08	0.04	–0.18	0.42	0.05	0.07	0.38	–0.10
Standard error	0.09	0.16	0.06	0.09	0.17	0.05	0.18	0.32	0.11	0.14	0.34	0.09
Minimum	–2.05	–2.24	–1.90	–1.77	–2.59	–0.98	–1.87	–0.97	–0.88	–0.88	–1.61	–0.45
Maximum	1.66	2.42	0.72	1.13	2.08	0.84	0.53	2.86	0.94	1.10	2.87	0.62
Count	42	42	42	40	40	40	15	15	15	12	12	12
Lower 95% CI	–0.25	–0.37	–0.14	–0.19	–0.53	–0.06	–0.68	0.09	–0.26	–0.21	–0.09	–0.20
Upper 95% CI	0.11	0.25	0.09	0.17	0.13	0.15	0.04	1.33	0.19	0.36	1.25	0.16

Currently, there is plain radiographic follow-up for 53 implants at 6 months, 52 at 1 year, 50 at 2 years, 18 at 5 years, 14 at 7 years, and 3 implants at 10 years (Appendix 1; see Supplementary data). Cortical thinning occurred in zones 1–12, but mainly in the distal zones at the 5-year follow-up (Figure 6C). Cancellization occurred in zones 1–12, and was most prominent at the 2-year follow-up in the middle zones (Figure 6D). Trabecular streaming appeared in only 3 of 52 implants in the proximal zones above the implant (zones 1 and 6) at the 1-year follow-up, but this number continued to rise over the course of the study (Figure 6E). Distal and endosteal resorption occurred only in zones 10 and 11, and none of the implants showed any resorption by 5 years (Appendix 1). For illustration purposes, the 2-year data are presented for each incidence since this is the time point with the greatest amount of implant follow-up (Figure 6A-E).

## Discussion

This is the first prospective RSA study to evaluate osseointegrated prostheses for use in transfemoral amputee patients. It is widely accepted that loosening of an orthopedic implant is one of the most common reasons for failure of the device. However, the RSA analysis for the OPRA system indicated that there was no statistically significant migration of the osseointegrated implant to this point. Graphically, some implants appeared to be rotating more than in the rest of the cohort. The 2 implants that showed the greatest degree of rotation (almost 3 degrees) had very high condition numbers in the RSA analysis. This “motion” could most likely be attributed to unreliable RSA analyses, since the median condition number for these patients was much higher (226) than the median condition number of the rest of the cohort (53). These high rotations occurred around the longitudinal axis. For geometric reasons, these have poorer resolution than rotations about the transverse and sagittal axes with this type of implant and radiographic set-up.

The other notable rotations occurred in the early follow-up period before 2 years. Early migration (for up to 6 months) of cementless stems in total hip arthroplasty is compatible with rigid fixation during the following postoperative years (Campbell et al. 2011). Long-term follow-up is necessary to definitively determine whether these implants will continue to rotate or whether the early motion encourages settling of the implant. The plain radiographic results of the implants with early rotation did not show any greater incidence of bone remodeling than in those with minimal rotation.

Söderberg et al. (2003) reported on efforts to minimize the motion between the exoprosthesis and the residual limb through a tighter connection between the two. While this study documented reduced rotation compared to standard socket prostheses, it was still substantially greater than the motion typically associated with an osseointegrated implant. Although some implants showed slight initial motion, the implants had stabilized at the 5-year follow-up. Of the 3 implants that failed, the motion detected using RSA was only slightly greater than the median degree of motion in the rest of the cohort.

The plain radiographic analysis of this cohort yielded results consistent with stress-shielding of fully coated femoral stems used in total hip arthroplasty. Stress-shielding has been reported with fully coated implants in total hip arthroplasty over the last 25 years, and the outcome of using these implants has not been compromised by this remodeling (Engh et al. 1992, Glassman et al. 2006). Engh et al. (1987) reported that moderate bone loss in patients with fully coated femoral stems did not affect implant stability or bony ingrowth. Similarly, the bone remodeling surrounding the implant that occurred in our study also did not compromise implant fixation or performance.

Unlike the case described by Wik et al. (2010) where a patient with a femoral condyle endoprosthesis experienced a fracture as a result of extreme bone resorption, the patients in our study with an osseointegrated prosthesis were not subjected to such significant stress shielding as a result of the implant. Wik et al. (2010) attributed this bone loss to the implant being fully coated and also to its large diameter. The majority of the patients' radiographs from our study showed only minimal amounts of bone remodeling around the implant, and ultimately this bone remodeling did not compromise implant fixation or performance. The OPRA system may have performed better as a result of surface structure of the implant, or of a more gradual rehabilitation program. However, even the cases that experienced more moderate bone loss did not show any indication of loosening or implant failure and they also had satisfactory clinical results.

This study had a few weaknesses, including the use of both analog and digital films for RSA follow-up. Several of the analog films were misplaced before the scanning process and we lost follow-up of some patients as a result. Careful attention was paid to the scanning of the films; thus, there was no difference in resolution—and ultimately no difference in the RSA kinematics between the analog and digital films. There was also the weakness that duplicate examinations were not performed to test the accuracy of the RSA set-up. Even so, the low mean errors of all the films suggest consistency in the set-up over the follow-up intervals. Finally, we could have provided a more in-depth analysis of the failed implants if an RSA film had been taken just before implant removal. There was only a film for the latest follow-up before the failure, and the kinematics did not necessarily indicate loosening or substantial migration.

There are several distinct advantages to using the OPRA system over the use of a conventional socket prosthesis (Brånemark et al. 2001, Hagberg et al. 2008). The transcutaneous nature of the OPRA system permits easy attachment and removal of the artificial limb through a quick-release mechanism. Ease of proper attachment also eliminates discomfort from wearing a limb that is improperly fitted. Similarly, since the skin-to-prosthesis interface is minimized and since the dermatological problems often associated with prosthesis attachment occur less frequently; we only had 1 superficial infection per patient every 2 years. Tranberg et al. (2011) showed that osseointegrated prostheses resulted in significant improvement in hip flexion and extension. While the use of osseointegrated prostheses does not fully restore the mechanics of a normal gait, they do improve the gait pattern and may help ameliorate lower back pain. Although there have been reports of pain and discomfort, osseointegrated implants result in increased mobility and fewer joint alignment problems after several years of use (Hagberg et al. 2005). The OPRA system provides a solution for patients who are unsuitable candidates for a conventional socket prosthesis, due either to amputation that has been at too high a level or to damage to the stump that has been too severe to allow fitting of a socket prosthesis.

Although there are clear advantages to the bone-anchored system, further development and refinement of the device and the surgical technique will be necessary in order to optimize the clinical results and to minimize the risk of complications. Such developmental work is in progress. In the future, a web-based registry will include current and future patients, providing more detailed outcome studies. The OPRA system has proven to be an attractive alternative to conventional socket prostheses, especially in the case of very high transfemoral amputations. This manner of attachment has significantly increased prosthesis use and has improved the quality of life of patients with transfemoral amputations (Hagberg et al. 2008). This RSA and radiographic analysis of the OPRA system has indicated that there is no substantial motion of the implant up to 7 years after the S2 procedure. The OPRA system is a promising approach for addressing the challenges faced by patients with transfemoral amputations.

## Supplementary data

Appendix is available at our website (www.actaorthop.org), identification number 4963.
