# Supplementary material for: Stable fixation of an osseointegated implant system for above-the-knee amputees: Titel RSA and radiographic evaluation of migration and bone remodeling in 55 cases
Source: Acta Orthop. 2012 Apr 24;83(2):121–8. doi: 10.3109/17453674.2012.678799 (PMC3339524; doi:10.3109/17453674.2012.678799)
Supplement: Supplementary file 1 [file ORT-1745-3674-83-121-s4963.pdf]

Supplementary article data

Stable fixation of an osseointegated implant system for above-the-knee amputees

Titel RSA and radiographic evaluation of migration and bone remodeling in 55 cases

Audrey K Nebergall[1], Charles R Bragdon[1], Anne Antonellis[1], Johan Kärrholm[2], Rickard Brånemark[2], Örjan Berlin[2], and Henrik Malchau[1]

[1]Harris Orthopaedic Laboratory, Massachusetts General Hospital, Boston, MA, USA; [2]Sahlgrenska University Hospital, Göteborg, Sweden.
Correspondence: hmalchau@partners.org
Submitted 11-06-24. Accepted 12-02-04

Supplementary data

Plain radiograph results and follow-up bone remodeling

A	B	C	D	E	F
Resorption					
A	0 (53)	0 (52)	0 (50)	0 (18)	0 (14)
B	0 (53)	0 (52)	0 (50)	0 (18)	0 (14)
C	0 (53)	0 (52)	1 (47)	0 (17)	0 (14)
D	0 (53)	0 (52)	0 (47)	0 (17)	0 (14)
1	0 (53)	0 (52)	0 (50)	0 (18)	0 (14)
2	0 (53)	0 (52)	0 (50)	0 (18)	0 (14)
3	0 (53)	0 (52)	0 (50)	0 (18)	0 (14)
4	0 (53)	0 (52)	0 (50)	0 (18)	0 (14)
5	0 (53)	0 (52)	0 (50)	0 (18)	0 (14)
6	0 (53)	0 (52)	0 (50)	0 (18)	0 (14)
7	0 (53)	0 (51)	0 (47)	0 (17)	0 (14)
8	0 (53)	0 (51)	0 (47)	0 (17)	0 (14)
9	0 (53)	0 (51)	0 (47)	0 (17)	0 (14)
10	0 (53)	1 (51)	2 (47)	0 (17)	0 (14)
11	0 (53)	1 (51)	1 (47)	0 (17)	0 (14)
12	0 (53)	0 (51)	0 (51)	0 (17)	0 (14)
Cortical thinning					
1	1 (53)	2 (52)	0 (50)	0 (18)	1 (14)
2	5 (53)	5 (52)	6 (50)	2 (18)	1 (14)
3	10 (53)	9 (52)	12 (50)	5 (18)	3 (14)
4	8 (53)	8 (52)	7 (50)	4 (18)	1 (14)
5	4 (53)	4 (52)	2 (50)	2 (18)	1 (14)
6	1 (53)	0 (52)	1 (50)	0 (18)	0 (14)
7	2 (52)	2 (51)	1 (47)	1 (17)	1 (14)
8	2 (52)	8 (51)	6 (47)	3 (17)	1 (14)
9	9 (52)	16 (51)	11 (47)	6 (17)	5 (14)
10	6 (52)	3 (51)	1 (47)	2 (17)	1 (14)
11	0 (52)	2 (51)	1 (47)	2 (17)	0 (14)
12	0 (52)	1 (51)	0 (47)	0 (17)	0 (14)

A	B	C	D	E	F
Cancellization					
A	0 (53)	0 (52)	2 (50)	0 (18)	1 (14)
B	0 (53)	0 (52)	1 (50)	0 (18)	1 (14)
C	0 (52)	0 (52)	0 (47)	0 (17)	1 (14)
D	0 (52)	0 (52)	0 (47)	0 (17)	1 (14)
1	1 (53)	2 (52)	1 (50)	2 (18)	1 (14)
2	3 (53)	8 (52)	12 (50)	4 (18)	6 (14)
3	4 (53)	9 (52)	14 (50)	3 (18)	5 (14)
4	5 (53)	7 (52)	15 (50)	3 (18)	4 (14)
5	5 (53)	5 (52)	15 (50)	2 (18)	2 (14)
6	2 (53)	2 (52)	5 (50)	1 (18)	2 (14)
7	0 (52)	0 (51)	3 (47)	1 (17)	1 (14)
8	3 (52)	5 (51)	10 (47)	5 (17)	4 (14)
9	4 (52)	6 (51)	9 (47)	5 (17)	4 (14)
10	6 (52)	9 (51)	14 (47)	3 (17)	6 (14)
11	3 (52)	9 (51)	16 (47)	4 (17)	4 (14)
12	1 (52)	3 (51)	9 (47)	3 (17)	3 (14)
Trabecular streaming					
1	0 (53)	1 (52)	3 (50)	6 (18)	6 (14)
6	0 (53)	2 (52)	9 (50)	6 (18)	8 (14)
7	0 (53)	0 (51)	2 (47)	4 (17)	5 (14)
12	0 (53)	0 (51)	1 (47)	3 (17)	3 (14)

A Zone
B 6-month: films with remodeling (films graded)
C 1-year: films with remodeling (films graded)
D 2-year: films with remodeling (films graded)
E 5-year: films with remodeling (films graded)
F 7-year: films with remodeling (films graded)

Open Access - This article is distributed under the terms of the Creative Commons Attribution Noncommercial License which permits any noncommercial use, distribution, and reproduction in any medium, provided the source is credited.
DOI 10.3109/17453674.2012.678799
